# Draft Genome Sequence of an Anicemycin Producer, *Streptomyces* sp. TP-A0648

**DOI:** 10.1128/genomeA.01468-16

**Published:** 2017-01-12

**Authors:** Hisayuki Komaki, Akira Hosoyama, Akane Kimura, Natsuko Ichikawa, Yasuhiro Igarashi

**Affiliations:** a Biological Resource Center, National Institute of Technology and Evaluation (NBRC), Chiba, Japan; b NBRC, Tokyo, Japan; c Biotechnology Research Center and Department of Biotechnology, Toyama Prefectural University, Toyama, Japan

## Abstract

We report the draft genome sequence of *Streptomyces* sp. TP-A0648 isolated from a leaf of *Aucuba japonica*. This strain produces a new tumor cell growth inhibitor designated anicemycin. The genome harbors at least 12 biosynthetic gene clusters for polyketides and nonribosomal peptides, suggesting the potential to produce diverse secondary metabolites.

## GENOME ANNOUNCEMENT

In our screening program of new antitumor compounds from microbial secondary metabolites, we isolated *Streptomyces* sp. TP-A0648 from a leaf of *Aucuba* *japonica* Thunb. collected in Toyama prefecture, Japan and discovered anicemycin as an inhibitor of anchorage-independent growth of human ovary cancer SKOV-3 cells from its culture broth. Anicemycin consists of four partial structures adenine, aminosugar, glycine, and fatty acid (1), and is structurally related to spicamycin (2) and septacidin (3). To assess the potential of the strain to produce other secondary metabolites such as polyketides and nonribosomal peptides, we carried out genome sequencing of *Streptomyces* sp. TP-A0648.

*Streptomyces* sp. TP-A0648 was deposited into the NBRC culture collection (NBRC 110465). The whole genome of *Streptomyces* sp. TP-A0648 was read using a combined strategy of shotgun sequencing with GS FLX+ (Roche; 86.6 Mb sequences, 11.3-fold coverage) and pair-end sequencing with MiSeq (Illumina; 729.9 Mb sequences, 95.2-fold coverage). These reads were assembled using Newbler v2.8, and subsequently finished using GenoFinisher (4), which led to a final assembly of 28 scaffolds and seven contig sequences of >500 bp each. The total size of the assembly was 7,621,989 bp, with a G+C content of 72.8%. Coding sequences were predicted by Prodigal (5). Polyketide synthase (PKS) and nonribosomal peptide synthetase (NRPS) gene clusters were analyzed in the same manner reported previously (6). This genome contains at least two type I PKS gene clusters, one type II PKS gene cluster, two type III PKS gene clusters, four NRPS gene clusters, and three hybrid PKS/NRPS gene clusters.

Type I PKS gene clusters in scaffold00001 and scaffold00020 showed high sequence similarities to biosynthetic gene clusters for annimycins (7) and C-1027 (8), respectively, although that in scaffold00001 is not completely sequenced. A type II PKS gene cluster in scaffold00001 was annotated to be for alnumycin biosynthesis (9). Type III PKS gene clusters in scaffold00008 and scaffold00009 are likely responsible for biosyntheses of phenolic lipids and 1,3,6,8-tetrahydroxynaphthalene, respectively, based on the similarity to SrsA (10) and RppA (11). NRPS gene clusters in scaffold00005, scaffold00009, scaffold00017, and scaffold000023 are predicted to synthesize a dipeptide composed of serine and proline; a siderophore including salicylate, threonine, and ornithine; a tripeptide comprising lysine, serine, and glycine; and an alanine-derived compound, respectively. The hybrid PKS/NRPS gene cluster in scaffold00008 resembles the biosynthetic gene cluster for polycyclic tetramate macrolactams of *S. griseus* NBRC 13350 (12). The remaining two hybrid PKS/NRPS gene clusters in scaffold00001 and scaffold00010 encode three modules, and displayed no significant similarities to the gene clusters whose products are characterized.

The genome analysis suggests the potential of *Streptomyces* sp. TP-A0648 to produce diverse polyketides and nonribosomal peptides. Further detailed inspection of the genome data may disclose the biosynthetic genes for anicemycin, for which the biosynthetic mechanisms remain unknown.

### Accession number(s).

The draft genome sequence of *Streptomyces* sp. TP-A0648 has been deposited in the DDBJ/ENA/GenBank database under the accession no. BDHJ00000000. The version described in this paper is the first version, BDHJ01000000.
